# The Antioxidant Potential of Resveratrol from Red Vine Leaves Delivered in an Electrospun Nanofiber System

**DOI:** 10.3390/antiox12091777

**Published:** 2023-09-18

**Authors:** Magdalena Paczkowska-Walendowska, Andrzej Miklaszewski, Bożena Michniak-Kohn, Judyta Cielecka-Piontek

**Affiliations:** 1 Department of Pharmacognosy and Biomaterials, Poznan University of Medical Sciences, Rokietnicka 3, 60-806 Poznan, Poland; jpiontek@ump.edu.pl; 2 Faculty of Mechanical Engineering and Management, Institute of Materials Science and Engineering, Poznan University of Technology, 60-965 Poznan, Poland; andrzej.miklaszewski@put.poznan.pl; 3 Department of Pharmaceutics, Ernest Mario School of Pharmacy, Rutgers—The State University of New Jersey, Piscataway, NJ 08899, USA; michniak@pharmacy.rutgers.edu; 4 Center for Dermal Research, Rutgers—The State University of New Jersey, Piscataway, NJ 08899, USA

**Keywords:** red vine leaf extract, antioxidant properties, anti-inflammatory properties, electrospun nanofibers, dissolution, permeability

## Abstract

Despite the wide pharmacological action of polyphenols, their usefulness is limited due to their low oral bioavailability, which is due to their low solubility and rapid first-pass metabolism. Red vine leaf extract is an herbal medicine containing several polyphenols, with resveratrol and polydatin as the main compounds exhibiting antioxidant and anti-inflammatory properties. In the first stage of the work, using the Design of Experiment (DoE) approach, the red vine leaf extract (50% methanol, temperature 70 °C, and three cycles per 60 min) was obtained, which showed optimal antioxidant and anti-inflammatory properties. In order to circumvent the above-described limitations and use innovative technology, electrospun nanofibers containing the red vine leaf extract, polyvinylpyrrolidone (PVP), and hydroxypropyl-β-cyclodextrin (HPβCD) were first developed. The optimization of the process involved the time of system mixing prior to electrospinning, the mixture flow rate, and the rotation speed of the collector. Dissolution studies of nanofibers showed improved resveratrol release from the nanofibers (over five-fold). Additionally, a PAMPA-GIT assay confirmed significantly better buccal penetration of resveratrol from this nanofiber combination (over ten-fold). The proposed strategy for electrospun nanofibers with the red vine leaf extract is an innovative approach to better use the synergy of the biological action of active compounds present in extracts that are beneficial for the development of nutraceuticals.

## 1. Introduction

Resveratrol belongs to the group of polyphenols with a stilbene structure [1]. The interest of scientists in resveratrol is fully justified because, in numerous in vitro studies as well as based on the results of clinical trials, its anti-obesity, cardioprotective, anticancer, antitumor, antidiabetic, antioxidant, anti-age effects, and glucose metabolism activities have been linked to its use [2].

A significant limitation in using the health-promoting properties of resveratrol is its low oral bioavailability. It is limited by both poor solubility and the hepatic first-pass effect [3]. To better use the potential of resveratrol, numerous studies are being conducted for the development of delivery systems to overcome this limitation. There is literature on developing electrospun nanofibers to enhance the characteristics of polyphenols, particularly resveratrol and its stilbene derivatives. While poly(caprolactone) nanoencapsulation has been reported for sustained release of resveratrol [4] for tissue engineering and wound healing applications [5], PVP-based nanofibers have been shown to increase resveratrol solubility [6,7]. Additionally, resveratrol was shielded from the unfavorable stomach pH situation and released into the intestines at a controlled rate by electrospun nanofibers containing zein [8]. Finally, the possibility of improving the dissolution and membrane permeation properties of resveratrol from electrospun nanofibers with resveratrol-rich extract from *Polygoni cuspidati* radix has also been demonstrated [9], where hydroxypropyl-2-cyclodextrin played a significant role as a solubilizer, apart from the very structure of the nanofibers.

However, it is worth noting that, apart from resveratrol, other polyphenolic compounds in plant materials have similar health-promoting properties. It is also known that the pro-health properties of a combination of these compounds together with resveratrol are stronger, and the systems of compounds with antioxidant potential may show synergy. Therefore, searching for ways to improve the solubility and, consequently, the bioavailability of natural compounds in the plant matrix is fully justified [10]. This approach is often followed and leads to a wider use of these compounds both in the industry of dietary supplements, cosmetics, as well as health foods.

One of the resveratrol-rich plant materials is *Vitis vinifera* and its fruit (grapes), and grape seeds are the richest sources of phenolic compounds, including resveratrol, and multi-directional pro-health activity is associated with them [11]. On the other hand, the leaves, which are somewhat wasted in fruit production, are an underestimated source of resveratrol and other polyphenols such as anthocyanins, flavonols, or phenolic acid derivatives [12,13,14].

Therefore, the optimization of the composition of the vine leaf extract in order to obtain the maximum extraction efficiency of resveratrol and polydatin, carried out for the first time in the presented work, allows the best use of the synergy of the coexistence of compounds present in the tested plant material. The second part of our research was directed at developing innovative systems in the form of electrospun nanofibers. This is the first use of the innovative electrospinning technology for vine leaf extracts to improve the solubility of resveratrol and, consequently, improve the bioavailability for the system of polyphenols present in the vine leaf extract. The Design of Experiments (DoE) approach was used to logically design the research study, including both parts, the extraction, and electrospinning processes. The proposed approach allowed for a better understanding of the processes, with fewer experiments than the case-by-case approach [15]. 

## 2. Materials and Methods

### 2.1. Plant Material

Plant material, red vine leaves, was purchased as a commercially available material from L’Herbier de France (France).

### 2.2. Chemicals and Reagents

Resveratrol (≥99%, HPLC; RSV) and polydatin (≥95%, HPLC) were obtained from Sigma-Aldrich (Poznan, Poland). Carriers such as (2-Hydroxypropyl)-β-cyclodextrin (HPβCD) average Mw ~1460 was supplied from Sigma-Aldrich (Poznan, Poland), and polyvinylpyrrolidone (PVP) as Kollidon^®^ 30 from BASF Pharma (Burgbernheim, Germany). Sigma-Aldrich (Poznan, Poland) provided reagents like 2,2-Diphenyl-1-picrylhydrazyl (DPPH), K_2_S_2_O_8_, 2,2′-Azino-bis(3-ethylbenzothiazoline-6-sulfonic acid) diammonium salt (ABTS, C_18_H_24_N_6_O_6_S_4_), neocuproine, C_2_H_7_NO_2_, CuCl_2_·H_2_O, CH_3_COONa·3H_2_O, 2,4,6-tris(2-pyridyl)-1,3,5-triazine (TPTZ, C_18_H_12_N_6_), FeCl_3_·6H_2_O, NaCl, bovine serum, hexadecyltrimethylammonium bromide (CTAB), hyaluronic acid (HA) for activity assays, KCl, NaCl, K_2_HPO_4_, MgCl_2_, CaCl_2_ and xylitol for dissolution studies, and mucin from porcine stomach for mucoadhesive assay. Prisma™ HT buffer, Acceptor Sink Buffer, and GIT lipid solution were obtained from Pion Inc., whereas HPLC grade acetonitrile and water were obtained from Merck. High-quality pure water and ultra-high-quality pure water were prepared using a Direct-Q 3 UV Merck Millipore purification system.

### 2.3. Extraction Process for the Red Vine Leaf Extract and Investigation of Its Biological Activity 

A factor experiment plan for three independent variables with three tiers of values (3^2^ complete factorial design) was created using the Design of Experiments (DoE) method. The composition of the extraction mixture, its temperature, and the processing duration were chosen as independent parameters (Table 1).

Total phenolic compound content, antioxidant activity (DPPH scavenging assay), and anti-inflammatory activities expressed as inhibition of hyaluronidase activity were chosen as the measures to assess extraction efficiency.

The total content of phenolic components was determined by using the method described previously [16]. Briefly, to each vial extract or gallic acid solution (25 µL), distilled water (200 µL), Folin–Ciocalteu reagent (15 µL), and 20% Na_2_CO_3_ solution (60 µL) were added. At 600 rpm, the plate was shaken for five minutes and then incubated in the dark for an additional 25 min at room temperature. At 760 nm, absorbance was measured. (Multiskan GO 1510, Thermo Fisher Scientific, Vantaa, Finland.) The total gallic acid content in the extracts was determined and reported as milligrams of gallic acid equivalents (GAE) per 1 g of plant material.

While antioxidant activity was determined by using an assay with 2,2-Diphenyl-1-picrylhydrazyl (DPPH), 2,2-Azino-bis(3-ethylbenzothiazoline-6-sulfonic Acid) (ABTS) Radical Cation-Based Assays, a Cupric Ion Reducing Antioxidant Capacity (CUPRAC) Assay, and a Ferric Ion Reducing Antioxidant Parameter (FRAP) Assay. All procedures were described previously [16]. In the case of DPPH, briefly, 25 μL of extract and 175 μL of 0.2 mmol/L DPPH solution were combined. During the 30 min of incubation in the dark, the mixture was continuously stirred. Absorbance was measured at 517 nm in comparison to the blank sample (25 μL of the extraction mixture and 175 μL of methanol) (Multiskan GO 1510, Thermo Fisher Scientific, Vantaa, Finland).

The previously established turbidimetric approach was used to determine the process of hyaluronidase inhibition [16]. Briefly, by mixing incubation buffer (50 mM, pH 7.0, with 77 mM NaCl and 1 mg/mL of bovine albumin; 25 µL), hyaluronidase enzyme (30 U/mL; 25 µL), extract (10 µL), and acetate buffer (pH 4.5; 15 µL), the sample was prepared. After 10 min of incubation at 37 °C, hyaluronic acid solution (0.3 mg/mL; 25 µL) was added and incubated for the next 45 min. After adding CTAB (200 µL) and waiting 10 min at room temperature, the turbinance was measured at 600 nm (Multiskan GO 1510, Thermo Fisher Scientific, Vantaa, Finland).

At this stage, a high-performance liquid chromatography method has been developed and validated to simultaneously determine both resveratrol and polydatin content. The stationary phase was a LiChrospher RP-18 column (5 μm, 250 mm × 4 mm) (Merck, Germany). The mobile phase was formic acid 0.1% (A) and acetonitrile (B) in gradient flow: 0–5 min, 10–17% B; 5–12 min, 17–18% B; 12–22 min, 18–22% B; 22–30 min, 22–33% B; 30–40 min, 33–35% B; 35–40 min, 10% B, with a constant mobile phase flow of 1.0 mL/min. The column temperature was set to 30 °C. At *λ*_max_ max = 310 nm, the detection was accomplished.

### 2.4. Obtaining Electrospun Nanofibers Containing Red Vine Leaf Extract

The NS + NanoSpinner Plus Electrospinning Equipment (Inovenso Ltd., Istanbul, Turkey) was used for the electrospinning operation. All nanofibers contained the same qualitative and quantitative composition, i.e., extract (5.0 mL), PVP (2.0 g), HPβCD (2.0 g), and methanol (5.0 mL). Based on data from the Design of Experiment (DoE) and a 3^2^ full factorial design experimental plan, process parameters, including mixture preparation time prior to electrospinning, mixture flow rate, and collector rotation speed, were chosen and are shown in Table 1. Process effectiveness, resveratrol content, the total amount of released bioactive, its permeability, and bioadhesion properties of the systems were the criteria utilized to measure the DoE process response.

First, 5.0 mL of the methanol and W10-optimized red vine leaf extract were mixed. After that, 2.0 g of HPβCD was added, and the mixture was well-stirred using a magnetic stirrer. Next, 2.0 g of PVP was added, and it was well incorporated using a magnetic stirrer. The solution was then placed into a syringe and electrospun at the following parameters: a voltage of 27 kV, flow rate as shown in Table 2, and distance of 12 cm. Aluminum foil-covered rotary collectors were used to collect the nanofibers. Optimized conditions have been set in light of preliminary tests.

The efficiency of the process was assessed by calculating the weight percentage of the produced nanofibers with the mass of the initial components.

### 2.5. Characterization Studies of the Electrospun Nanofibers

#### 2.5.1. Scanning Electron Microscopy (SEM)

SEM was used to see the nanofiber’s surface morphology. After being gold-palladium sputter coated, the nanofibers were observed using a Quanta 250 FEG, FE scanning electron microscope.

#### 2.5.2. X-ray Diffraction (XRPD)

By using the copper anode (CuK—1.54 Å) in a Brag-Brentano reflection mode configuration with 45 kV and 40 mA parameters, an X-ray diffraction (XRD, Panalytical Empyrean, Almelo, The Netherlands) apparatus was used to study the crystalline structure of the samples. The measurement settings were always set to 3–60° with a 45 s step between each degree.

#### 2.5.3. Fourier Transform Infrared Spectroscopy with Attenuated Total Reflectance (FTIR-ATR) 

The FTIR-ATR spectra were measured between 400 cm^−1^ and 4000 cm^−1^, with a resolution set to 1 cm^−1^, with a Shimadzu IRTracer-100 spectrometer equipped with a QATR-10 single bounce–diamond extended range and LabSolution IR software. 

### 2.6. Characterization of Electrospun Nanofiber’s Functionality 

#### 2.6.1. Resveratrol Release and Permeability Assays

Using the chromatography technique, the rate of dissolution of resveratrol incorporated into nanofibers and its penetration through membrane systems simulating the walls of the gastrointestinal tract after release from nanofibers were tested. While the spectroscopic technique was used to assess the antioxidant potential of resveratrol after its introduction into the structure of nanofibers. 

A dissolving tool called the Agilent 708-DS was used to conduct dissolution tests on electrospun nanofibers. A conventional basket technique was used at 37 ± 0.5 °C and 50 rpm for stirring. Nanofibers were added to 300 mL of synthetic saliva (pH 6.8; potassium chloride 1.20 g, sodium chloride 0.85 g, dipotassium hydrogen orthophosphate 0.35 g, calcium chloride 0.20 g, xylitol 20.0 g, and water up to 1 L; pH was adjusted to 6.8 by 1 M HCl). At certain intervals, liquid samples were taken, and an equivalent volume of temperature-stabilized medium was substituted. A nylon membrane filter with a mesh size of 0.45 µm was used to filter the samples. The above-mentioned HPLC technique was used to measure the levels of resveratrol in the filtrated acceptor solutions. The acquired active compound release patterns were fitted to the zero-order, first-order, Higuchi, and Korsmeyer–Peppas models in order to examine the release kinetics [17].

The permeability of resveratrol encapsulated in nanofibers via synthetic biological membranes was investigated using the PAMPA^TM^ (parallel artificial membrane permeability assay) gastrointestinal tract (GIT) assay from Pion Inc. Nanofibers were dissolved using donor solutions, which were artificial saliva solutions with a pH of 6.8. The Donor solutions (artificial saliva solution at pH 6.8) were used to dissolve nanofibers. Acceptor Prisma buffer with a pH of 7.4 was added to the acceptor plates. The plates were assembled and incubated for 15 min at 37 °C while continuously stirring at 50 rpm. At least three times each experiment was rerun. The HPLC technique mentioned above was used to determine the quantity of penetrated resveratrol.

The apparent permeability coefficients (*P_app_*) were calculated from the following equation:

$$P_{app}=\frac{-ln\left(1-\frac{C_{A}}{C_{equilibrium}}\right)}{S\times \left(\frac{1}{V_{D}}+\frac{1}{V_{A}}\right)\times t}$$

where *V_D_*—donor volume, *V_A_*—acceptor volume, *C_equilibrium_*—equilibrium concentration $C_{equilibrium}=\frac{C_{D}\times V_{D}+C_{A}\times V_{A}}{V_{D}+V_{A}}$, *C_D_*—donor concentration, *C_A_*—acceptor concentration, *S*—membrane area, *t*—incubation time (in seconds).

#### 2.6.2. Nanofibers’ Activity

Electrospun nanofibers’ antioxidant activity was tested using the DPPH assay covered in Section 2.3.

#### 2.6.3. Mucoadhesive Properties

The bond strength between mucin and polymers for bioadhesion was measured using a viscometric technique. The assessment was completed using the previously specified methodology [18]. The increased intermolecular frictional force per unit area, known as the force of bioadhesion *F*, was calculated as follows:

*F =* (*η_t −_ η_m_*_*−*_*η_p_*) × *σ*

where *η_t_* is the nanofibers’ viscosity coefficient, and *η_m_* is mucin’s viscosity coefficient, *η_p_* is PVP/HPβCD’s viscosity coefficient, and *σ* is the rate of shear per second. 

### 2.7. Statistical Analysis

Software called Statistica 13.3 was used for the statistical analysis. To check if the data were normal, the Shapiro–Wilk test was applied. The variances between the mean values were investigated using the ANOVA test and Tukey’s post hoc range test for multiple comparisons. Differences across groups were considered significant at *p* < 0.05. Correlations were examined using principal component analysis (PCA) with PQStat Software version 1.8.4.142 (2022).

## 3. Results and Discussion

### 3.1. Obtaining and Characterization of Biological Activity of Red Vine Leaf Extract

So far, there is little information on the impact of individual parameters of the vine leaves extraction process. As a result, a number of extracts were created utilizing a design methodology that focused on evaluating the impact of input elements (such as temperature, the makeup of the extraction mixture, and extraction duration) on the extract’s attributes (such as its amount of active ingredients and biological activity). A complete factorial design model was developed in this study to evaluate the effectiveness of the extraction procedure (Table 1). The efficiency mentioned above of the extraction process was evaluated by determination of total polyphenol content, antioxidant activity (measured by four methods: DPPH, ABTS, CUPRAC, FRAP), and anti-inflammatory activity measured as hyaluronidase enzyme inhibition (Table 3; Table S1, Supplementary Material).

**Figure 1:**
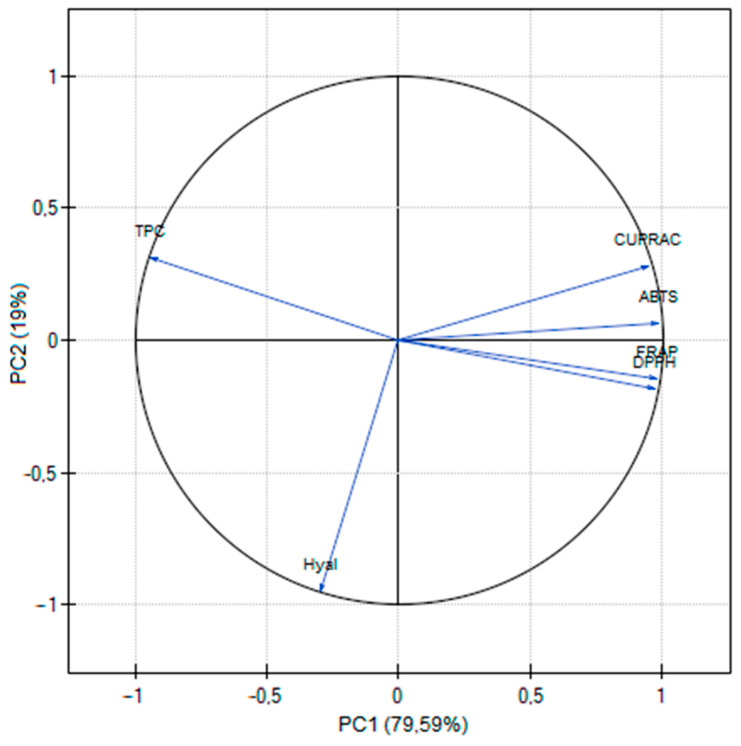
PCA for the phytochemical characterization of extracts.

Using the DPPH radical, the activity of resveratrol alone was assessed, which was IC_50_ = 0.0257 µg/mL, whereas for vitamin C (positive control) IC_50_ = 0.0097 µg/mL. Looking at the results of the antioxidant activity (Table 3), some differences can be noticed between the tests used. This is due to different mechanisms for assessing activity. DPPH and ABST used different radicals [19], whereas the CUPRAC assay is based on the reduction of Cu(II) to Cu(I) in the presence of bis(neocuproine), which then chelate to Cu(I); whereas FRAP monitors the reduction of iron (III) to iron (II) in the presence of 2,4,6-tris-(2-pyridyl)-s-triazine (TPTZ) [20]. However, there is a positive correlation between all methods (Figure 1), also widely described in the literature [21].

In the next step, all outputs were statistically analyzed. Based on the Pareto diagram (Figure S1, Supplementary Material), it was found that the extraction temperature was a statistically significant factor affecting the total phenolic content. This effect had a value with a positive sign, i.e., with the increase in the extraction temperature, the TPC increased. 

In the case of the antioxidant activity (Figure S2, Supplementary Material) and anti-inflammatory effect (Figure S3, Supplementary Material), both the percentage of methanol in the extraction mixture and the temperature were statistically significant factors affecting the IC_50_. In this case, values possessed a negative sign; that is, as the starting values increased, the IC_50_ decreased, which meant an increase in overall activity. The antioxidant properties of red wine made from grapes have been known for decades, associated with their ability to scavenge synthetic and endogenous free radicals [22]. However, the health-promoting properties of vine leaf preparations should also be related to the presence of polyphenols, including stilbene analogs such as resveratrol and polydatin [23], as well as flavonols such as quercetin and derivatives [24].

All obtained results were statistically analyzed using principal component analysis (PCA). PC1 was positively correlated with antioxidant activity and negatively associated with total polyphenol content and anti-inflammatory activity. PC2 was favorably connected with total phenolic compounds and adversely correlated with anti-inflammatory chemicals (Figure 1). Additionally, a robust negative correlation was shown between the total content of polyphenols and antioxidant activity (Figure 1; Table S2, Supplementary Material).

It was feasible to choose the technical aspects of the extraction process that produced an extract with the greatest qualities and the maximum activity based on the findings of the study and statistical analysis. Analysis of the importance of the input components revealed those with a positive sign (TPC) (Figure S4a, Supplementary Material) and with a negative sign (antioxidant and anti-hyaluronidase activities) (Figure S4b, Supplementary Material). Based on the utility contour profiles model, it was feasible to estimate the model and identify the optimal extraction process parameters, which were 50% methanol in the extraction mixture, 70 °C, and three cycles every 60 min (a statistically insignificant parameter). Optimized process parameters were used to prepare the E10 extract, the activity of which was determined to validate the model. TPC for E10 extract was 17.04 ± 0.29 mg GAE/1 g plant material, whereas antioxidant activity was measured by DPPH: IC_50_ = 61.64 ± 1.38 µg/mL, and the anti-inflammatory effect was measured by inhibition of the hyaluronidase enzyme: IC_50_ = 4.82 ± 0.25 mg/mL. The E10 extract was used in the second part of the experimental work, i.e., to prepare the electrospun nanofibers.

**Figure 2:**
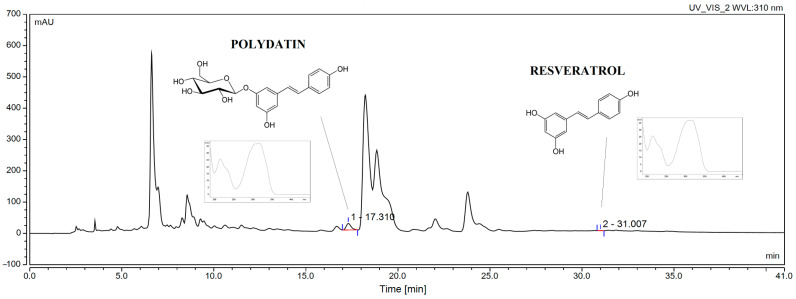
Chromatogram of optimized extract.

In parallel, with the newly developed methods it was possible to assess active compounds such as the polydatin and resveratrol (Figure 2). While data from the literature are available on the determination of stilbene derivatives in other parts of the grapevine, there are no data on the determination of the content of these compounds in leaf extracts. According to ICH criteria, the proposed HPLC technique was verified, and the validation parameters are listed in Table S3 (Supplementary Material).

### 3.2. Obtaining of Electrospun Nanofibers Containing Red Vine Leaf Extract and Identification of Their Structure

The second part of the work focused on optimizing the electrospinning process. Electrospinning parameters (applied electric field, needle-to-collector distance, flow rate, and needle diameter), solutions (solvent, polymer concentration, solution viscosity, and conductivity), and environmental parameters (relative humidity and temperature) are a few of the variables that influence the electrospinning process [25]. All of these factors directly impact the production of bead-free and smooth electrospun fibers [26]. Several of these parameters, such as the optimal composition of HPβCD and PVP, applied voltage (25 kV), and optimum tip-to-collector distance (12 cm) have been tested and selected based on previous studies [9]. Therefore, in the case of the presented studies, the aim was to assess the significance of the effect of the time of preparing the mixture and the parameters of the process itself, i.e., the mixture’s flow rate and the collector’s rotation. During the procedure, ambient parameters such as operating temperature and humidity were kept constant.

In the first stage, the efficiency of the process was assessed in terms of the amount of material obtained (Table 4; Table S4, Supplementary Material; Figure S5, Supplementary Material). It was noticed that with the increase in the flow rate of the mixture through the needle, the amount of obtained material decreases.

Further, the obtained electrospun nanofibers were characterized in terms of their morphology (SEM), structure (XRPD), and formation of potential intermolecular bonds (FTIR-ATR). 

**Figure 3:**
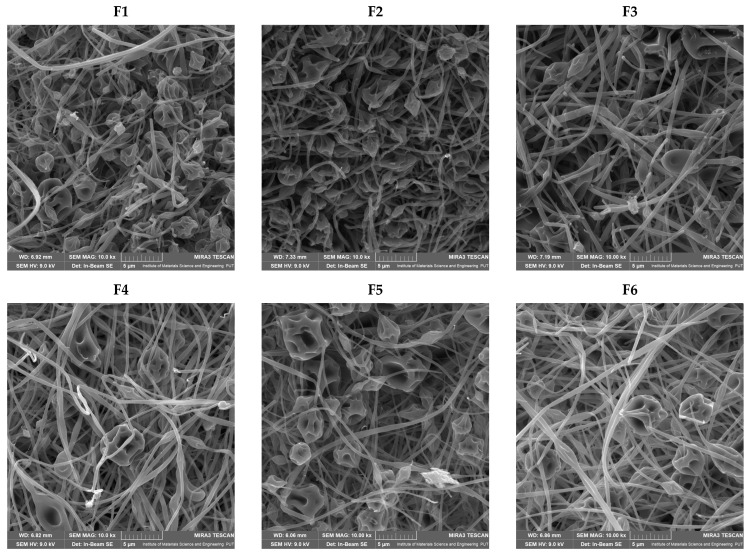

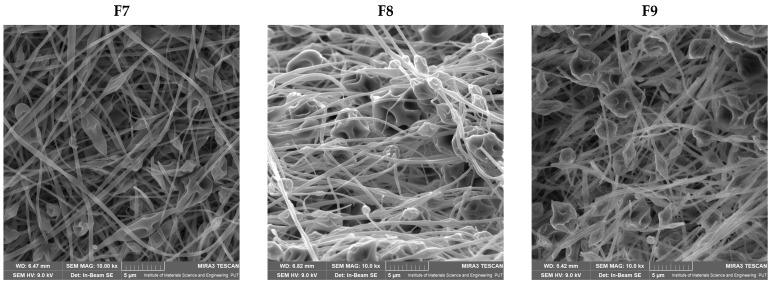
SEM images of electrospun nanofibers F1–F9.

SEM images for all systems confirmed the nanofiber structure (Figure 3). Most researchers and scientists agree that the distribution of fiber diameters is the crucial factor influencing how well electrospun materials function in their activity. So, the size of the obtained nanofibers (Table 5; Figure S6, Supplementary Material) and their appearance in terms of fiber shape were evaluated. There were no statistically significant relationships between the process parameters and the diameter of the nanofibers (Figure S6, Supplementary Material). However, it was noticed that with increasing mixing time, the number of beads in the nanofibers decreased (Figure 3). Since the mixing time of the mixture before the process does not affect the size of the nanofibers but improves their homogeneity, it is worth considering a 60 min mixing time.

**Figure 4:**
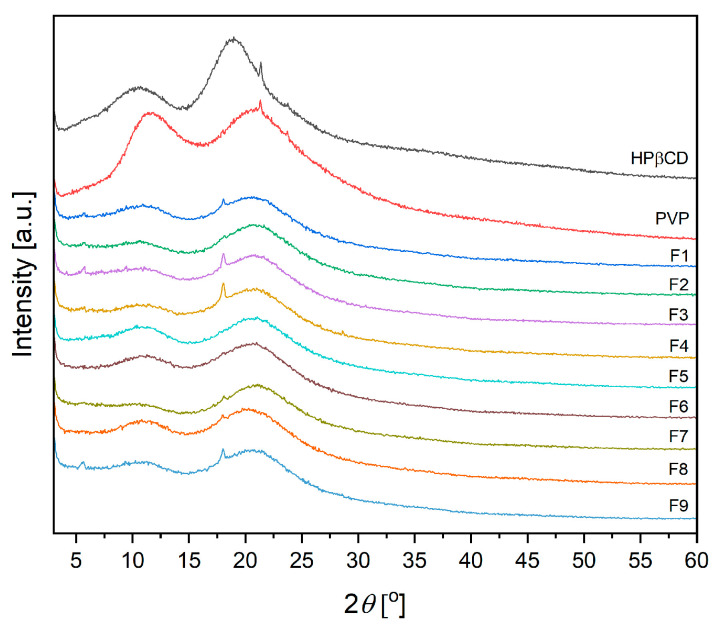
Diffractograms for components (HPβCD, PVP) and nanofibers F1–F9.

The starting materials (PVP, HPβCD) demonstrated a high widening of the diffraction peaks and, with low intensity, suggested the amorphous structure of these powders, according to the X-ray diffractograms (Figure 4) [9]. Moreover, all nanofibers are also characterized generally as being amorphous.

**Figure 5:**
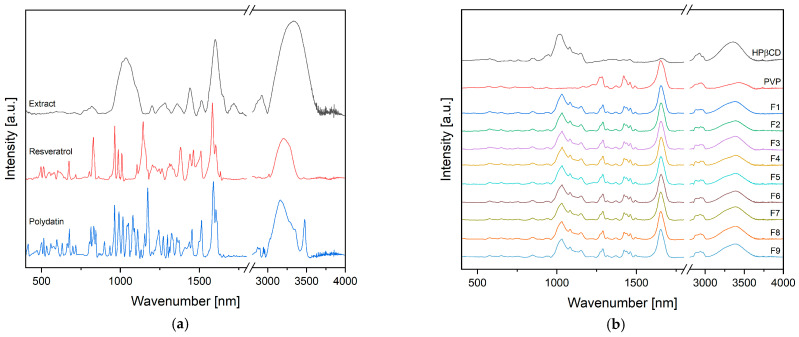
FTIR-ATR spectra for components (**a**) and nanofibers F1–F9 (**b**).

Finally, the intramolecular interactions between the extract, PVP, and HPβCD were analyzed using FTIR-ATR (Figure 5). From previous research, we knew that the given process parameters did not affect the interactions between the ingredients. It was essential to test if changes in the process could induce these interactions. Based on the spectra of F1–F9 nanofibers, it was found that they are the sum of individual components, and no significant changes in the position and intensity of individual bands were observed (Figure 4). This would suggest that there are no molecular interactions between the extract and the other components.

### 3.3. Characterization of Electrospun Nanofibers’ Functionality

The purpose of creating nanofibers was to improve the ingredients’ physicochemical properties, i.e., resveratrol’s solubility and permeability. Therefore, in this part of the work, the influence of process parameters on the physicochemical and pharmaceutical properties of the obtained form was assessed.

The content of resveratrol was the first factor to be examined, as shown in Table 6. None of the process parameters was statistically significant (Figure S7, Supplementary Material).

**Figure 6:**
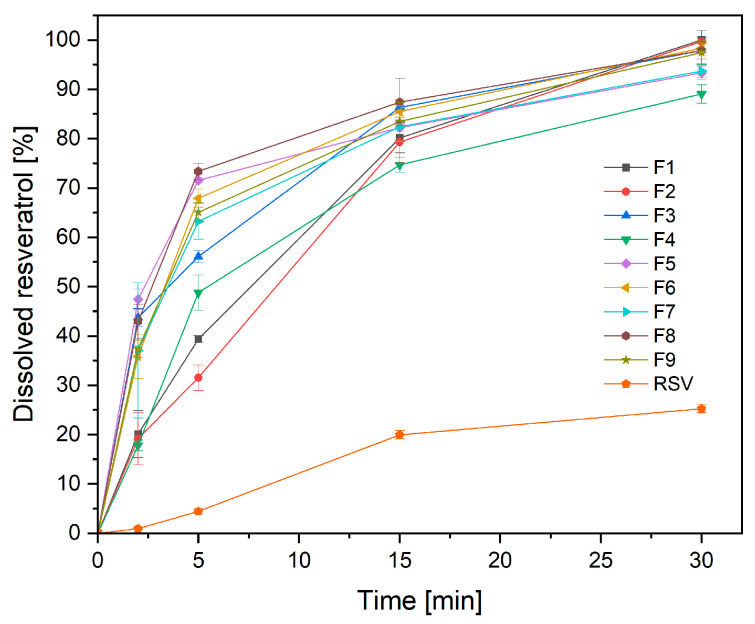
Dissolution profiles of resveratrol from the nanofibers F1–F9 at artificial saliva solution at pH 6.8 (*n* = 3).

A critical parameter that significantly affects the product’s effectiveness is the release of the active substance from the nanofibers (Figure 6, Table 7). For this purpose, a previously developed modified method using baskets was used [9]. When compared to a pure powder component, it was possible to increase resveratrol’s solubility rate by five times. In this case, the mixing time resulted in a significant increase in the percentage of resveratrol released at 5 min (Table 7; Figure S8, Supplementary Material). This is consistent with the previous microscopic evaluation of nanofibers, the absence of beads, and their greater homogeneity for nanofibers made from a long-mixed mixture. Regardless of the parameters of the process itself, the increase in resveratrol’s release rate was significant. The advantages of nanofiber creation, such as high load capacity, efficient encapsulation, and a high surface area to volume ratio, which can all improve the dissolution rate, can be used to explain this phenomenon [7]. The composition used to produce nanofibers is also important, because the HPβCD used showed an increase in the solubility of active compounds by high amorphization, wetting, solubilizing, and complexing properties of this cyclodextrin [27,28].

In addition, the kinetics of resveratrol release from the prepared nanofibers were evaluated (Table S5, Supplementary Material). The most likely release mechanism is Higuchi kinetics, suggesting that resveratrol release happens by diffusion across scattered vesicles. The second plausible mechanism is Korsmeyer–Peppas kinetics with n above 0.89, indicating kinetics approaching zero-order kinetics, which has also been demonstrated.

**Figure 7:**
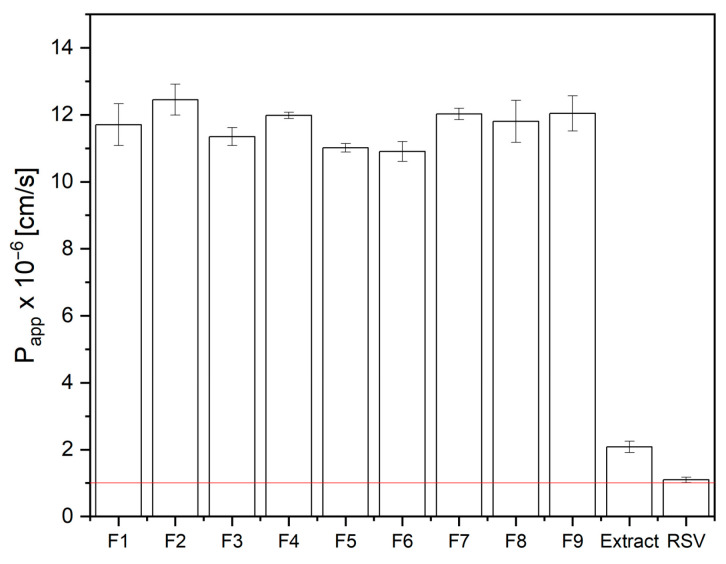
Apparent permeability coefficients for resveratrol standard, as well as resveratrol from extract and nanofibers F1–F9 (*n* = 3).

In addition, the oral mucosa are regarded as a desirable site for resveratrol administration due to the bypassing of the first-pass effect. As it has been shown, a significant increase in the rate of resveratrol release from electrospun nanofibers was obtained. This, in turn, can result in an increase in permeation through biological membranes. Therefore, using the PAMPA test, the permeability of resveratrol from the prepared extract and the nanofibers was assessed (Figure 7). No statistical influence of the electrospinning process parameters on this property was recorded (Figure S9, Supplementary Material).

By showing an increase in the rate of resveratrol release at oral pH and an increase in the compound’s permeability through the membrane simulating the mucosa, the obtained data can be extrapolated to assess the increase in bioavailability of resveratrol.

**Figure 8:**
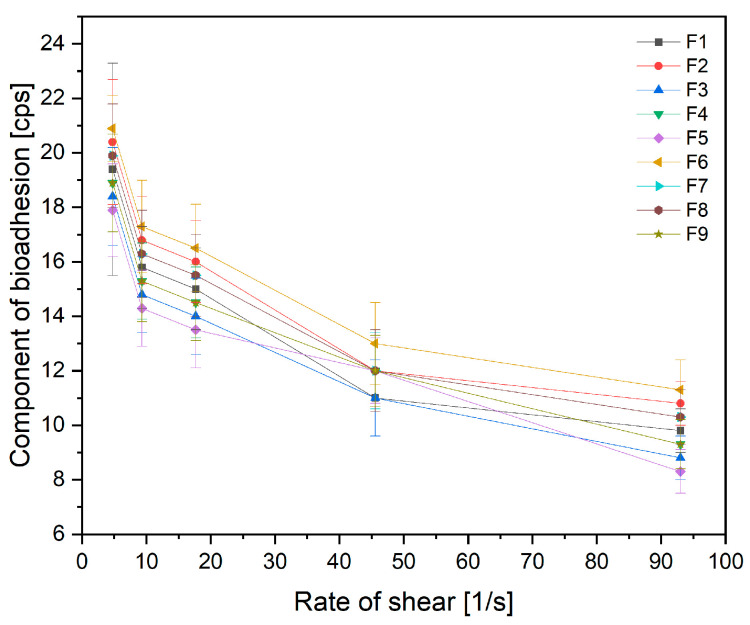
Component of bioadhesion of nanofibers F1–F9 (*n* = 3).

When evaluating the product in terms of the possibility of its use in the oral cavity, it is essential to keep the electrospun nanofibers within the oral cavity, precisely on the buccal layer for the precise time allowing the active compound to dissolve and penetrate. Thus, the rheological properties of the obtained electrospun nanofibers were assessed (Figure 8). No statistical influence of the electrospinning process parameters on the mucoadhesive effect was noted (Figure S10, Supplementary Material).

Finally, we tested if the antioxidant and anti-inflammatory effects of the extract were still present using the nanofibers created using the techniques outlined in the first section of the paper. Firstly, the results showed that the antioxidant and anti-inflammatory properties remained at a level comparable to those of the initial extract, which confirms the lack of a negative impact of the process on the biological activity of the final nanofibers (Table S6, Supplementary Materials). Secondly, no statistically significant influence of the process parameters on biological activity was demonstrated. 

Summing up all the relationships, it can be concluded that only the time of mixing the mixture before the process and the flow rate of the mixture were significant. While there is not much work on the effect of mixture preparation, the flow effect is widely reported in the literature. The flow of the polymeric solution via the needle tip determines the shape of the electrospun nanofibers. Beads may occur if the flow rate is increased beyond the critical point [26,29]. The current studies showed that better material quality is obtained after a longer mixing time and a lower flow rate.

**Figure 9:**
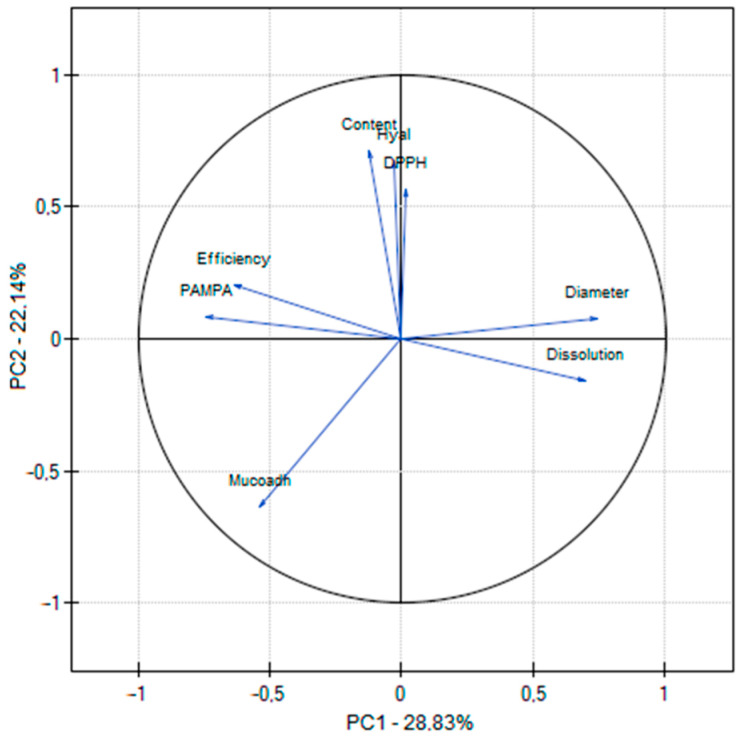
PCA for the characterization of the electrospinning process and obtained nanofibers.

All properties of the process and the obtained nanofibers were subjected to a correlation assessment using principal component analysis (PCA). A correlation was found between the nanofibers’ antioxidant and anti-inflammatory activity and the resveratrol content. The essence of the optimized process was also indicated because in the case where the efficiency of the process was higher, a smaller diameter of nanofibers was obtained (Figure 9; Table S6, Supplementary Material), which confirmed previous observations.

It was feasible to anticipate the statistical model and identify the ideal electrospinning process parameters using the completed research and statistical studies (Figure S11, Supplementary Material). The optimal parameters of the electrospinning process were found to be 60 min of mixing the mixture before the process, a mixture flow rate of 2 mL/h, and a collector rotation speed of 200 rpm. 

## 4. Conclusions

Using the DoE approach, it was possible to determine the optimal parameters of the vine leaves extraction process: 50% of methanol in the extraction mixture, temperature 70 °C, and three cycles per 60 min. The antioxidant and anti-inflammatory properties of the obtained extract have been demonstrated, indicating the potential of the plant material’s health-promoting properties.

The DoE approach was an effective technique to select the optimal electrospinning conditions. As a result, resveratrol-rich vine leaf extract-loaded PVP/HPβCD was successfully created for use as a novel, controlled drug delivery extract system. The data from the electrospun nanofibers with improved resveratrol solubility (over five-fold) and significantly better buccal penetration of resveratrol (over ten-fold) show the potential of buccal application to increase the bioavailability of resveratrol.

The described DoE approach provides a flexible and modular design platform whose scope and size can be expanded or decreased depending on each case’s complexity and design goals. The DoE approach was successfully used in this case to optimize the process of extracting plant material and prepare electrospun nanofibers containing the previously obtained extract. A DoE-like approach is therefore suitable for characterizing and understanding extraction and electrospinning procedures. In addition, this approach can also be potentially used to create any material utilizing any other cutting-edge manufacturing method.

## Figures and Tables

**Table 1:** Factorial Extraction Process Experiment Plan.

No.	% of Methanol in the Extraction Mixture	Temperature [°C]	Time [min]
E1	0	30	30
E2	0	50	90
E3	0	70	60
E4	35	30	90
E5	35	50	60
E6	35	70	30
E7	70	30	60
E8	70	50	30
E9	70	70	90

**Table 2:** Factorial Electrospinning Process Experiment Plan.

No.	Mixing Time [min]	Flow Rate of Mixture [mL/h]	Rotation Speed of Collector [rpm]
F1	5	1	100
F2	5	2	300
F3	5	3	200
F4	60	1	300
F5	60	2	200
F6	60	3	100
F7	115	1	200
F8	115	2	100
F9	115	3	300

**Table 3:** Total phenolic content, antioxidant, and anti-hyaluronidase activities of extracts E1–E9 (*n* = 6).

No.	TPC [mg GAE/1 g Plant Material]	Antioxidant Activity				Anti-Inflammatory Activity
		DPPH IC_50_ [µg/mL]	ABTS IC_50_ [µg/mL]	CUPRAC IC_0.5_ [µg/mL]	FRAP IC_0.5_ [µg/mL]	Inhibition of Hyaluronidase Activity IC_50_ [mg/mL]
E1	9.65 ± 0.23	124.61 ± 3.69	199.83 ± 15.37	508.09 ± 8.24	83.22 ± 7.92	4.73 ± 0.07
E2	10.91 ± 0.23	108.75 ± 13.25	180.61 ± 10.97	421.45 ± 38.54	74.39 ± 5.52	4.08 ± 0.08
E3	12.78 ± 0.36	94.47 ± 3.20	167.83 ± 10.02	312.97 ± 4.07	62.33 ± 4.18	3.63 ± 0.22
E4	13.50 ± 0.26	86.93 ± 3.22	143.14 ± 10.93	89.09 ± 6.20	53.38 ± 6.16	5.66 ± 0.87
E5	15.93 ± 0.41	67.89 ± 0.68	116.84 ± 26.53	75.69 ± 0.83	51.08 ± 4.25	5.51 ± 0.95
E6	16.84 ± 0.29	60.78 ± 2.33	113.37 ± 7.15	66.60 ± 6.55	45.28 ± 3.21	4.58 ± 0.39
E7	12.21 ± 0.63	95.47 ± 2.88	132.21 ± 25.28	69.88 ± 4.66	62.01 ± 2.70	9.12 ± 0.10
E8	13.76 ± 0.36	76.07 ± 6.64	131.60 ± 30.61	60.28 ± 3.41	54.47 ± 4.34	8.03 ± 0.39
E9	17.21 ± 0.65	62.79 ± 2.96	104.02 ± 22.23	48.00 ± 2.44	41.77 ± 3.44	5.17 ± 0.32

**Table 4:** Efficiency of electrospinning process.

F1	F2	F3	F4	F5	F6	F7	F8	F9
%								
62.42	63.58	50.92	60.76	61.81	58.35	62.45	61.93	51.98

**Table 5:** Diameters of nanofibers F1–F9.

F1	F2	F3	F4	F5	F6	F7	F8	F9
Fiber diameter [nm]								
321.87 ± 8.97	339.51 ± 7.45	515.87 ± 17.86	489.42 ± 13.31	441.47 ± 14.14	445.33 ± 15.47	462.96 ± 23.31	365.96 ± 7.88	365.96 ± 12.20

**Table 6:** Content of resveratrol in nanofibers F1–F9 (*n* = 3).

F1	F2	F3	F4	F5	F6	F7	F8	F9
Content [µg] in 100 mg of nanofibers								
0.306 ± 0.009	0.563 ± 0.042	0.338 ± 0.098	0.054 ± 0.001	0.751 ± 0.046	0.027 ± 0.001	0.387 ± 0.018	0.141 ± 0.011	0.190 ± 0.034

**Table 7:** Total amount of released resveratrol from nanofibers F1–F9 at 5 min (*n* = 3).

F1	F2	F3	F4	F5	F6	F7	F8	F9
Resveratrol dissolution in 5 min [%]								
39.33	31.51	56.06	48.77	71.55	67.88	63.20	73.35	65.08

## Data Availability

Data are contained within the presented article or supplementary material.

## Supplementary Materials

The following supporting information can be downloaded at: https://www.mdpi.com/article/10.3390/antiox12091777/s1, Table S1. Response data for extraction process (DoE). Figure S1. Statistical analysis for total phenolic content in extracts E1–E9: (a) Pareto plot of standardized effects for total phenolic content in extracts E1–E9; (b) Response surface plots presenting the dependence of methanol content in the extraction mixture and extraction temperature on the total phenolic content in extracts for constant time at level 60 min. Figure S2. Statistical analysis for antioxidant activity of extracts E1–E9 measured by DPPH method: (a) Pareto plot of standardized effects for antioxidant activity; (b) Response surface plots presenting the dependence of methanol content in the extraction mixture and extraction temperature on antioxidant activity of extracts for constant time at level 60 min. Figure S3. Statistical analysis for anti-inflammatory activity of extracts E1–E9: (a) Pareto plot of standardized effects for anti-inflammatory activity of extracts E1–E9; (b) Response surface plots presenting the dependence of methanol content in the extraction mixture and extraction temperature on anti-inflammatory activity of extracts for constant time at level 60 min. Table S2. Correlation matrix for extract properties. Figure S4. Prediction of the optimization model for obtaining extracts based on effect with positive signs like TPC (a) and those with negative signs like DPPH and hyaluronidase assays (b). Table S3. Validation parameters of HPLC method. Table S4. Response data for electrospinning process (DoE). Figure S5. Statistical analysis for process efficiency: (a) Pareto plot of standardized effects for viscosities of the prepared solutions for electrospinning; (b) Response surface plots presenting the dependence of flow rate and rotation speed on the efficiency of electrospinning process for mixing time at level 60 min. Figure S6. Statistical analysis for diameter of nanofibers: (a) Pareto plot of standardized effects for diameter of nanofibers F1–F9; (b) Response surface plots presenting the dependence of rotation speed and flow rate on the efficiency of electrospinning process for mixing time at level 60 min. Figure S7. Statistical analysis for resveratrol content: (a) Pareto plot of standardized effects for hesperidin content in nanofibers F1–F9; (b) Response surface plots presenting the dependence of mixing time and rotation speed on the resveratrol content for constant flow rate at level 2 mL/h. Figure S8. Statistical analysis for resveratrol dissolution: (a) Pareto plot of standardized effects for resveratrol dissolution in 5 min from nanofibers F1–F9; (b) Response surface plots presenting the dependence of mixing time and flow rate on the resveratrol dissolution for constant rotation speed at level 200 rpm. Table S5. Parameters of mathematical models fitted to the release profiles of nanofibers F1–F15. Figure S9. Statistical analysis for resveratrol permeability: (a) Pareto plot of standardized effects for resveratrol permeability from nanofibers F1–F9; (b) Response surface plots presenting the dependence of rotation speed and flow rate on the resveratrol permeability for constant mixing time at level 60 min. Figure S10. Statistical analysis for component of mucoadhesion: (a) Pareto plot of standardized effects for mucoadhesive properties of nanofibers F1–F9; (b) Response surface plots presenting the dependence of rotation speed and mixing time on the component of mucoadhesion for constant flow rate at level 2 mL/h. Table S6. Antioxidant and anti-inflammatory properties of nanofibers F1–F9. Table S7. Correlation matrix for electrospun nanofibers properties. Figure S11. Prediction of the optimization model for obtaining extracts based on effects with positive signs like process efficiency, resveratrol content, dissolution and permeability, as well as component of mucoadhesion (a) and this with negative signs like nanofibers diameters (b).
