# Supplementary material for: The Antioxidant Potential of Resveratrol from Red Vine Leaves Delivered in an Electrospun Nanofiber System
Source: Antioxidants (Basel). 2023 Sep 18;12(9):1777. doi: 10.3390/antiox12091777 (PMC10525167; doi:10.3390/antiox12091777)
Supplement: Supplementary file 1 [file antioxidants-12-01777-s001.zip › antioxidants-2593081-supplementary.pdf]

The Antioxidant Potential of Resveratrol from Red Vine Leaves Delivered in an Electrospun Nanofiber System

Supplementary material

Table S1. Response data for extraction process (DoE)

	Inputs			Outputs		
No.	% of methanol in the extraction mixture	Temperature	Time [min]	TPC [mg GAE/1g plant material]	Antioxidant activity DPPH IC_{50} [µg/mL]	Inhibition of hyaluronidase activity IC_{50} [mg/mL]
E1	0	30	30	9.65	124.61	4.73
E2	0	50	90	10.91	108.75	4.08
E3	0	70	60	12.78	94.47	3.63
E4	35	30	90	13.50	86.93	5.66
E5	35	50	60	15.93	67.89	5.51
E6	35	70	30	16.84	60.78	4.58
E7	70	30	60	12.21	95.47	9.12
E8	70	50	30	13.76	76.07	8.03
E9	70	70	90	17.21	62.79	5.17

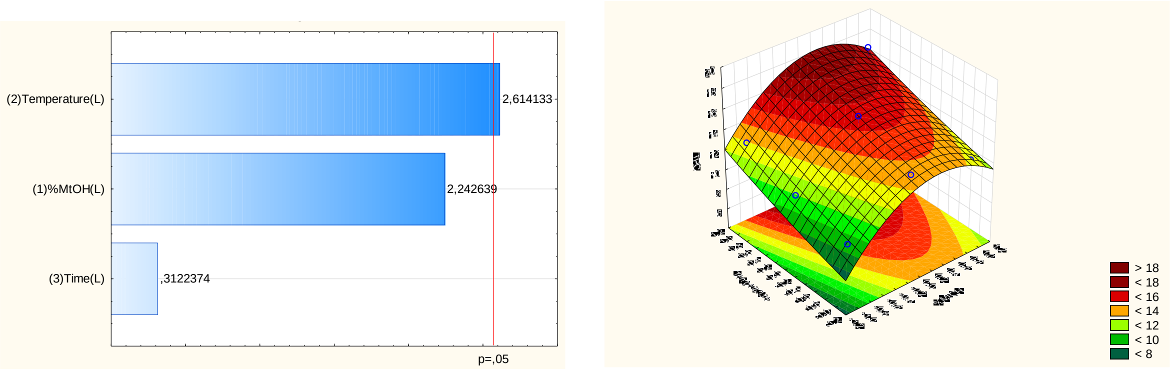

(a) (b)

Figure S1. Statistical analysis for total phenolic content in extracts E1-E9: (a) Pareto plot of standardized effects for total phenolic content in extracts E1-E9; (b) Response surface plots presenting the dependence of methanol content in the extraction mixture and extraction temperature on the total phenolic content in extracts for constant time at level 60 minutes.

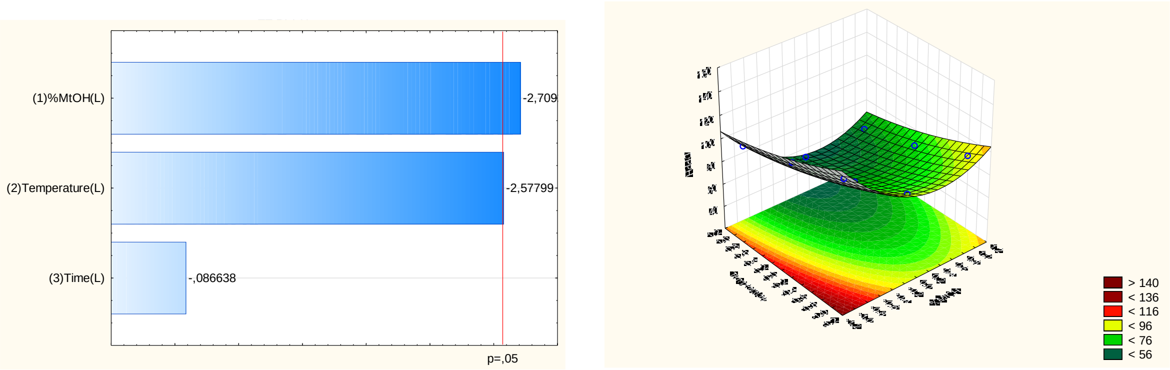

(a) (b)

Figure S2. Statistical analysis for antioxidant activity of extracts E1-E9 measured by DPPH method: (a) Pareto plot of standardized effects for antioxidant activity; (b) Response surface plots presenting the dependence of methanol content in the extraction mixture and extraction temperature on antioxidant activity of extracts for constant time at level 60 minutes.

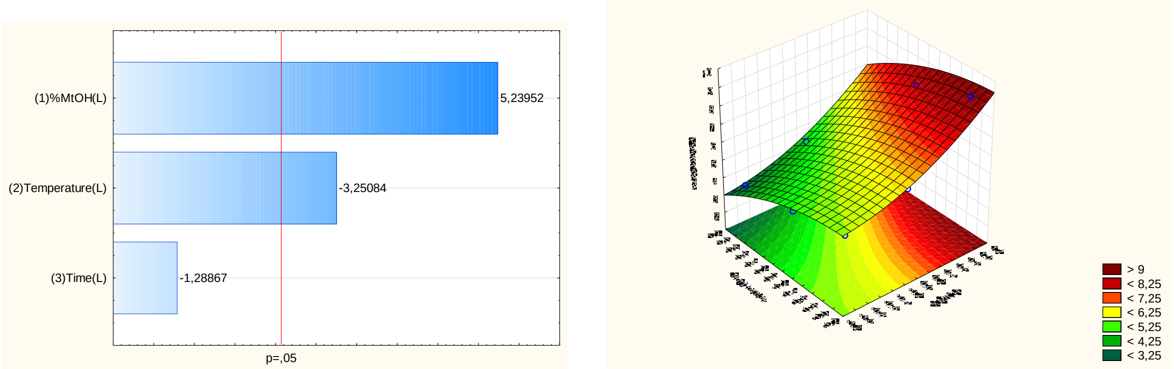

(a) (b)

Figure S3. Statistical analysis for anti-inflammatory activity of extracts E1-E9: (a) Pareto plot of standardized effects for anti-inflammatory activity of extracts E1-E9; (b) Response surface plots presenting the dependence of methanol content in the extraction mixture and extraction temperature on anti-inflammatory activity of extracts for constant time at level 60 minutes.

Table S2. Correlation matrix for extract properties

Variable	TPC	DPPH	ABTS	CUPRAC	FRAP	Hyal
TPC	1,0000	-0,9776	-0,9224	-0,7984	-0,9605	-0,0116
DPPH	-0,9776	1,0000	0,9485	0,8696	0,9750	-0,1228
ABTS	-0,9224	0,9485	1,0000	0,9490	0,9496	-0,3605
CUPRAC	-0,7984	0,8696	0,9490	1,0000	0,9044	-0,5407
FRAP	-0,9605	0,9750	0,9496	0,9044	1,0000	-0,1516
Hyal	-0,0116	-0,1228	-0,3605	-0,5407	-0,1516	1,0000

Statistically significant relationships are marked in red

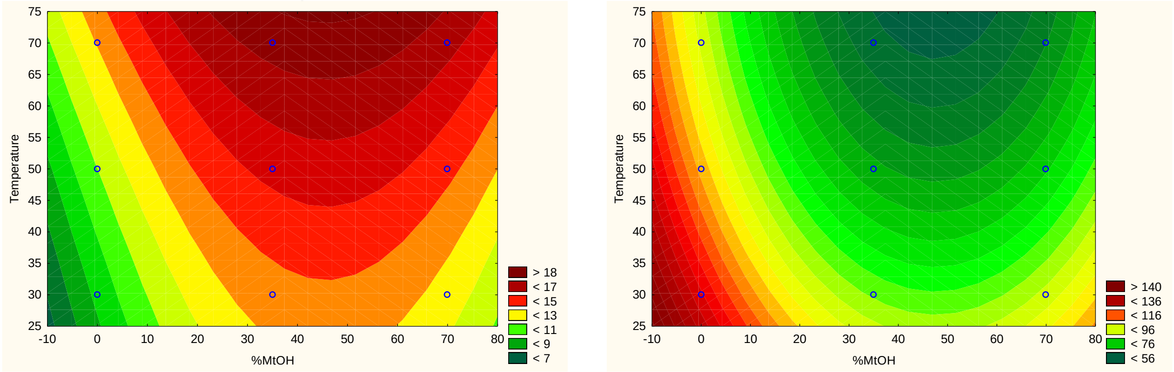

(a) (b)

Figure S4. Prediction of the optimization model for obtaining extracts based on effect with positive sign like TPC (a) and those with negative sing like DPPH and hyaluronidase assays (b).

Table S3. Validation parameters of HPLC method

Parameter	**Resveratrol**
Linearity: y = ax + b	
$a \pm S_a$	0.315 ± 0.003

b ± S_b	insignificant (α=0.05)
Correlation coefficient (r)	0.999
Range of linearity [µg/mL]	84.4–8440.0
Intra-day precision, RSD (<5% required) = repeatability	
The lowest concentration	1.83
The middle concentration	1.15
The highest concentration	1.68
Limit of detection (LOD) [µg/mL]	17.99
Limit of quantification (LOQ) [µg/mL]	54.50

Table S4. Response data for electrospinning process (DoE)

Inputs				Outputs					
**No.**	**Mixing time [min]**	**Flow rate of mixture [ml/h]**	**Rotation speed of collector [rpm]**	**Efficiency**	**Diameter of nanofibers**	**Content of resveratrol [ug] in 100 mg of nanofibers**	**Total amount of released resveratrol from nanofibers at 5 minutes**	**Resveratrol permeability**	**Component of mucoadhesion**
F1	5	1	100	62.42	321.87	0.306	39.33	11.71	15.00
F2	5	2	300	63.58	339.51	0.563	31.51	12.45	16.00
F3	5	3	200	50.92	515.87	0.338	56.06	11.35	14.00
F4	60	1	300	60.76	489.42	0.054	48.77	11.98	14.50
F5	60	2	200	61.81	441.47	0.751	71.55	11.02	13.50
F6	60	3	100	58.35	445.33	0.027	67.88	10.90	16.50
F7	115	1	200	62.45	462.96	0.387	63.20	12.02	15.50
F8	115	2	100	61.93	365.96	0.141	73.35	11.80	15.50
F9	115	3	300	51.98	365.96	0.190	65.08	12.04	14.50

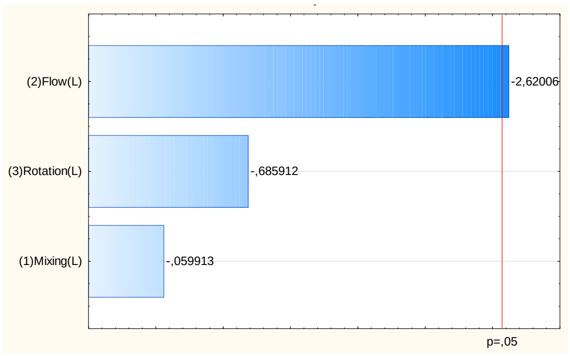

(a)

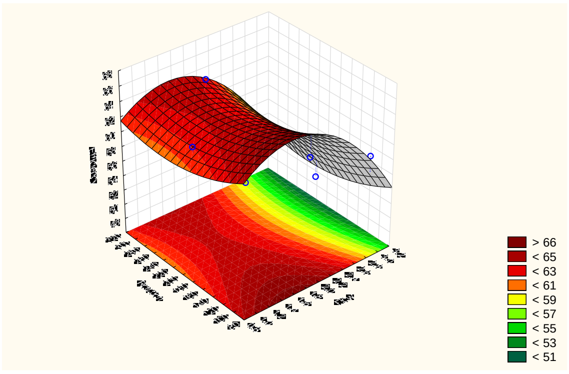

(b)

Figure S5. Statistical analysis for process efficiency: (a) Pareto plot of standardized effects for viscosities of the prepared solutions for electrospinning; (b) Response surface plots presenting the dependence of flow rate and rotation speed on the efficiency of electrospinning process for mixing time at level 60 minutes.

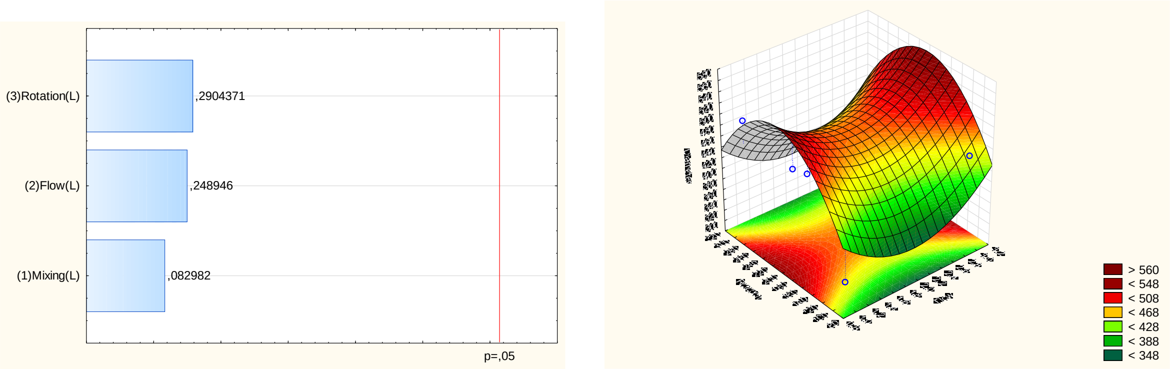

(a) (b)

Figure S6. Statistical analysis for diameter of nanofibers: (a) Pareto plot of standardized effects for diameter of nanofibers F1-F9; (b) Response surface plots presenting the dependence of rotation speed and flow rate on the efficiency of electrospinning process for mixing time at level 60 minutes.

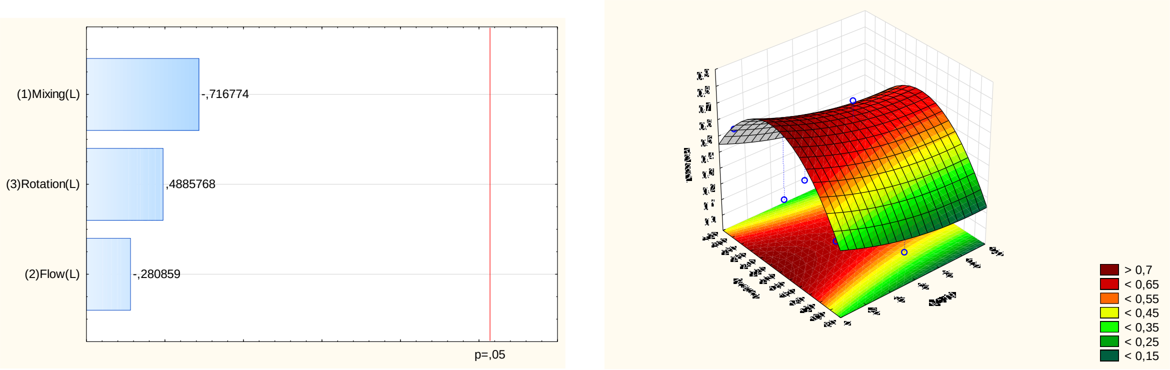

(a) (b)

Figure S7. Statistical analysis for resveratrol content: (a) Pareto plot of standardized effects for hesperidin content in nanofibers F1-F9; (b) Response surface plots presenting the dependence of mixing time and rotation speed on the resveratrol content for constant flow rate at level 2 ml/h.

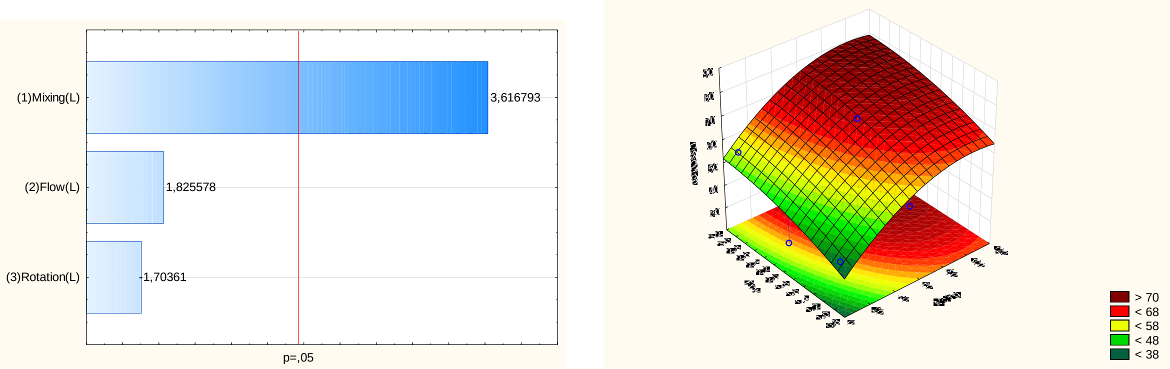

(a) (b)

Figure S8. Statistical analysis for resveratrol dissolution: (a) Pareto plot of standardized effects for resveratrol dissolution in 5 minutes from nanofibers F1-F9; (b) Response surface plots presenting the dependence of mixing time and flow rate on the resveratrol dissolution for constant rotation speed at level 200 rpm.

Table S5. Parameters of mathematical models fitted to the release profiles of nanofibers F1-F15

Formulation	Mathematical model							
No.	Zero-order kinetic		First-order kinetic		Higuchi kinetic		Korsmeyer-Peppas kinetic	
	K	R^2	K	R^2	K	R^2	R^2	n
F1	**190.56**	**0.90**	6.40	0.50	**21.71**	**0.94**	**0.88**	**1.05**
F2	**194.50**	**0.92**	6.54	0.54	**21.39**	**0.91**	**0.89**	**1.05**
F3	160.96	0.74	5.56	0.36	**21.73**	**0.96**	**0.79**	**1.04**
F4	163.98	0.82	6.13	0.47	**20.01**	**0.95**	**0.87**	**1.04**
F5	139.23	0.60	5.26	0.32	**21.07**	**0.93**	**0.77**	**1.04**
F6	161.76	0.70	5.26	0.32	**22.29**	**0.96**	**0.80**	**1.06**
F7	152.89	0.70	5.53	0.36	**21.19**	**0.96**	**0.80**	**1.04**
F8	152.95	0.64	5.44	0.34	**22.34**	**0.94**	**0.78**	**1.05**
F9	159.37	0.72	5.59	0.36	**21.87**	**0.96**	**0.80**	**1.05**
RSV	**54.31**	**0.92**	6.81	0.80	5.42	0.82	**0.86**	**0.76**

the most fitting mathematical model is shown in bold

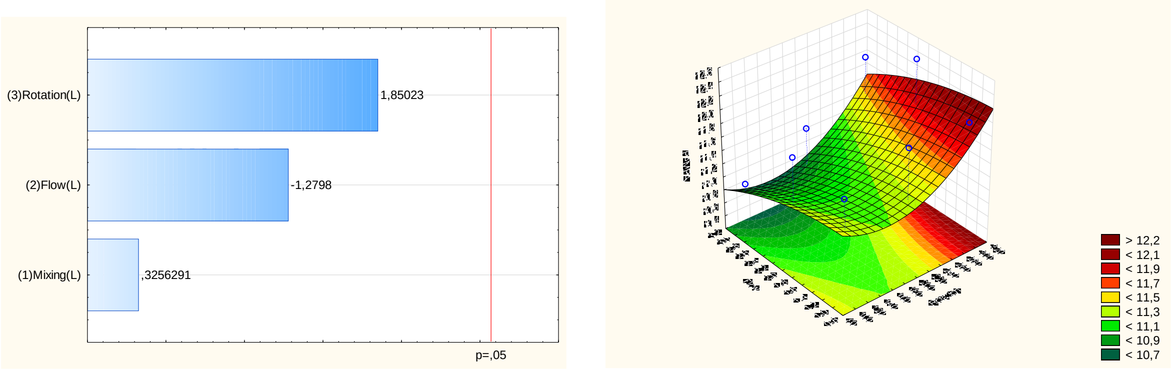

(a) (b)

Figure S9. Statistical analysis for resveratrol permeability: (a) Pareto plot of standardized effects for resveratrol permeability from nanofibers F1-F9; (b) Response surface plots presenting the dependence of rotation speed and flow rate on the resveratrol permeability for constant mixing time at level 60 minutes.

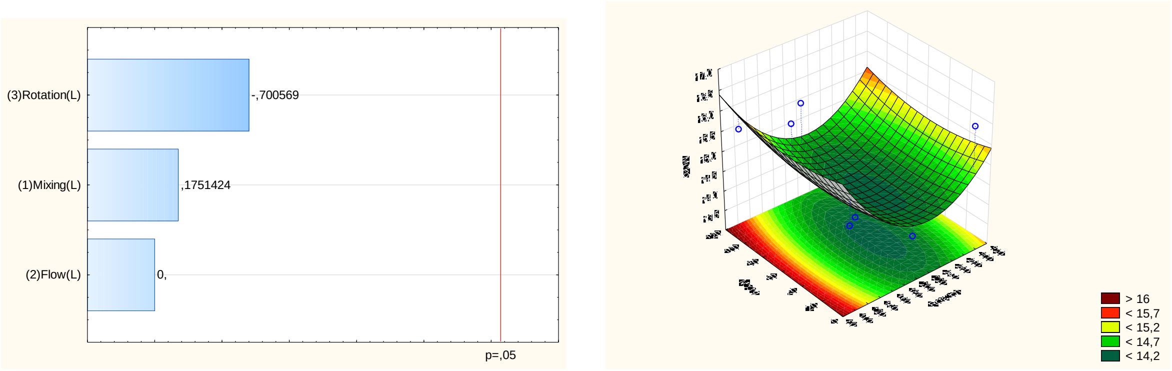

(a) (b)

Figure S10. Statistical analysis for component of mucoadhesion: (a) Pareto plot of standardized effects for mucoadhesive properties of nanofibers F1-F9; (b) Response surface plots presenting the dependence of rotation speed and mixing time on the component of mucoadhesion for constant flow rate at level 2 ml/h.

Table S6. Antioxidant and anti-inflammatory properties of nanofibers F1-F9

**No.**	**Antioxidant properties DPPH IC_{50} [mg/ml]**	**Anti-inflammatory properties Anti-hyaluronidase activity IC_{50} [mg/ml**
F1	4.01 ± 0.64	231.03 ± 2.34
F2	4.10 ± 0.63	241.35 ± 1.34
F3	3.82 ± 0.42	235.73 ± 1.92
F4	4.57 ± 0.01	238.63 ± 2.01
F5	4.33 ± 0.35	240.92 ± 1.73
F6	4.14 ± 0.03	233.94 ± 1.37
F7	3.90 ± 0.16	232.73 ± 2.03
F8	3.80 ± 0.05	238.82 ± 2.14
F9	3.88 ± 0.08	240.72 ± 2.52

Table S7. Correlation matrix for electrospun nanofibers properties

Variable	Efficiency	Diameter	Content of resveratrol	Dissolution of resveratrol	PAMPA	Component of mucoadhesion	DPPH	Hyal
Efficiency	1,0000	-0,3547	0,2803	-0,2503	0,2303	0,3639	0,3604	-0,0701
Diameter	-0,3547	1,0000	-0,1057	0,3261	-0,4201	-0,3407	0,2750	-0,1146
Content of resveratrol	0,2803	-0,1057	1,0000	-0,1508	-0,0016	-0,3890	0,0481	0,2982
Dissolution of resveratrol	-0,2503	0,3261	-0,1508	1,0000	-0,5543	-0,1642	-0,1930	0,0862
PAMPA	0,2303	-0,4201	-0,0016	-0,5543	1,0000	0,2113	-0,1296	0,2502
Component of mucoadhesion	0,3639	-0,3407	-0,3890	-0,1642	0,2113	1,0000	-0,1874	-0,2931
DPPH	0,3604	0,2750	0,0481	-0,1930	-0,1296	-0,1874	1,0000	0,2466
Hyal	-0,0701	-0,1146	0,2982	0,0862	0,2502	-0,2931	0,2466	1,0000

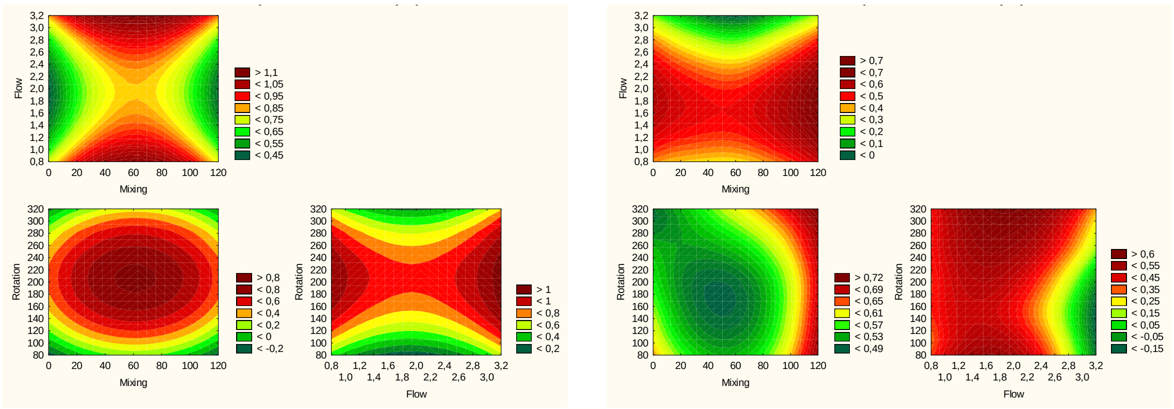

(a) (b)

Figure S11. Prediction of the optimization model for obtaining extracts based on effects with positive sign like process efficiency, resveratrol content, dissolution and permeability as well as component of mucoadhesion (a) and this with negative sing like nanofibers diameters (b).
